# Supplementary material for: Constructing a draft Indian cattle pangenome using short-read sequencing
Source: Commun Biol. 2025 Apr 13;8:605. doi: 10.1038/s42003-025-07978-0 (PMC11994783; doi:10.1038/s42003-025-07978-0)
Supplement: Supplementary file 1 — Supplementary information file [file 42003_2025_7978_MOESM1_ESM.pdf]

Constructing a Draft Indian Cattle Pangenome Using Short-Read Sequencing

Sarwar Azam[1,2], Abhisek Sahu[1], Naveen Kumar Pandey[1], Mahesh Neupane[3], Curtis P Van Tassell[3], Benjamin D Rosen[3,*], Ravi Kumar Gandham[1], Subha Narayan Rath[2], Subeer S Majumdar[1,*]

[1]National Institute of Animal Biotechnology, Hyderabad, India
[2]Indian Institute of Technology Hyderabad, Sangareddy, India
[3]Animal Genomics and Improvement Laboratory, USDA-ARS, Beltsville, MD 20705, USA

[*]Corresponding author: Email- ben.rosen@usda.gov; subeer@niab.org.in

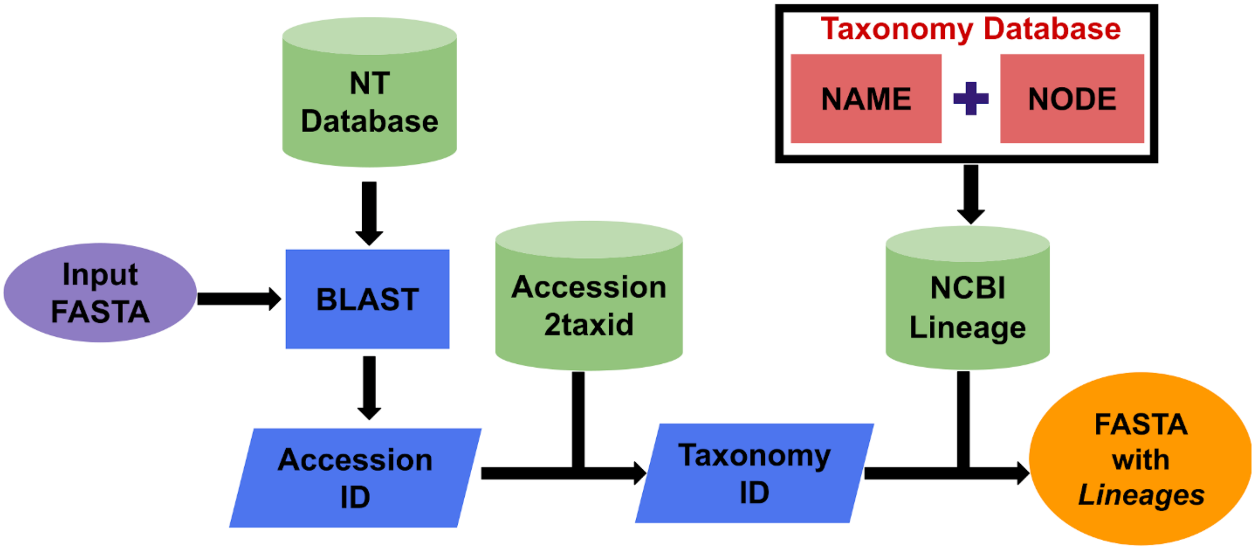

Supplementary Figure 1: Flowchart outlines the computational workflow of the Fasta2Lineage tool, primarily implemented in Bash scripting.

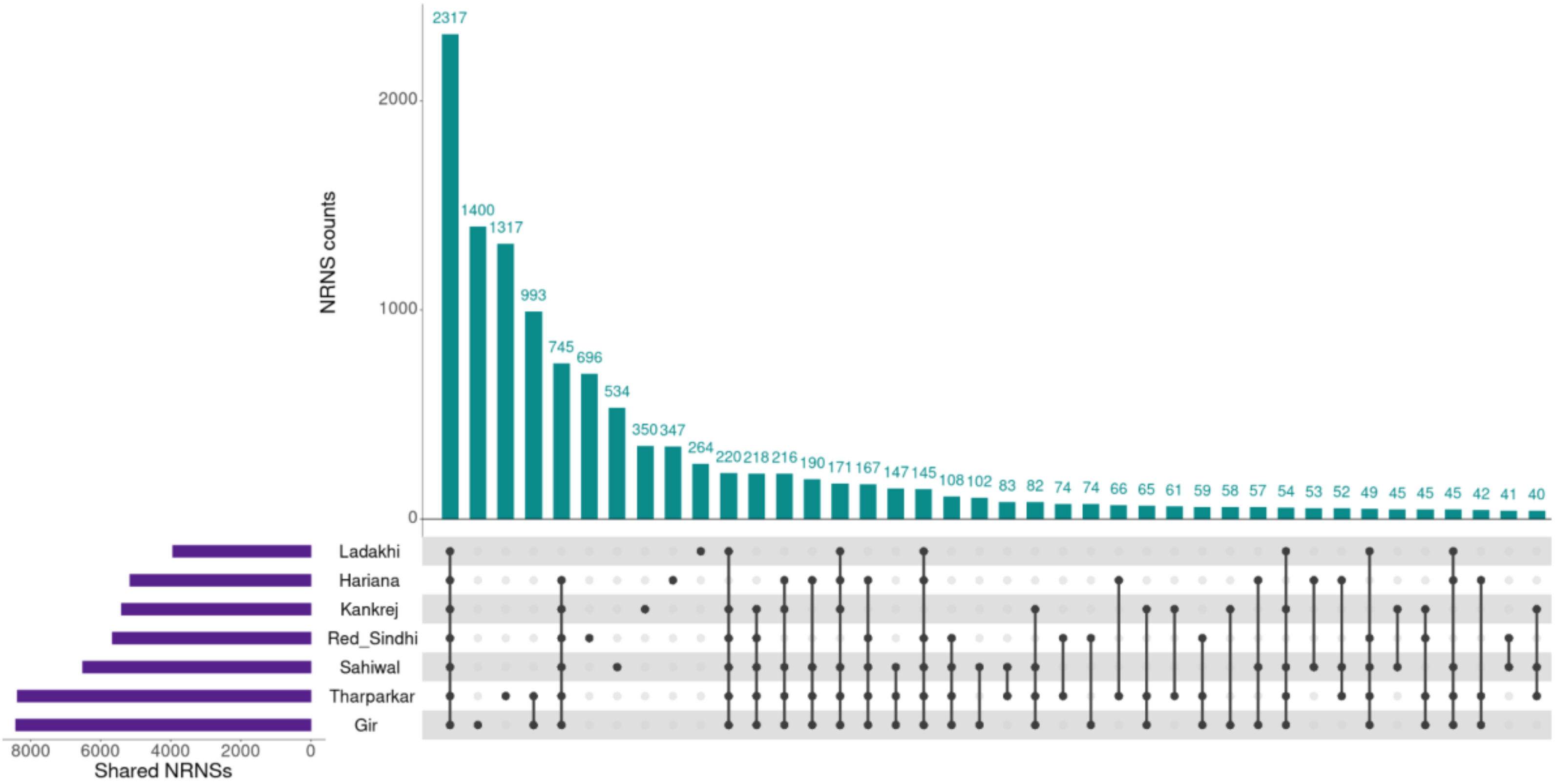

Supplementary Figure 2: Overlap of NRNS among *Bos indicus* breeds. An UpSet plot illustrating the shared NRNS among seven *Bos indicus* breeds identified using the PanGA pipeline. Each vertical bar represents a combination of breeds, with the height indicating the number of shared NRNS. The horizontal bars display the count of these shared NRNSs.

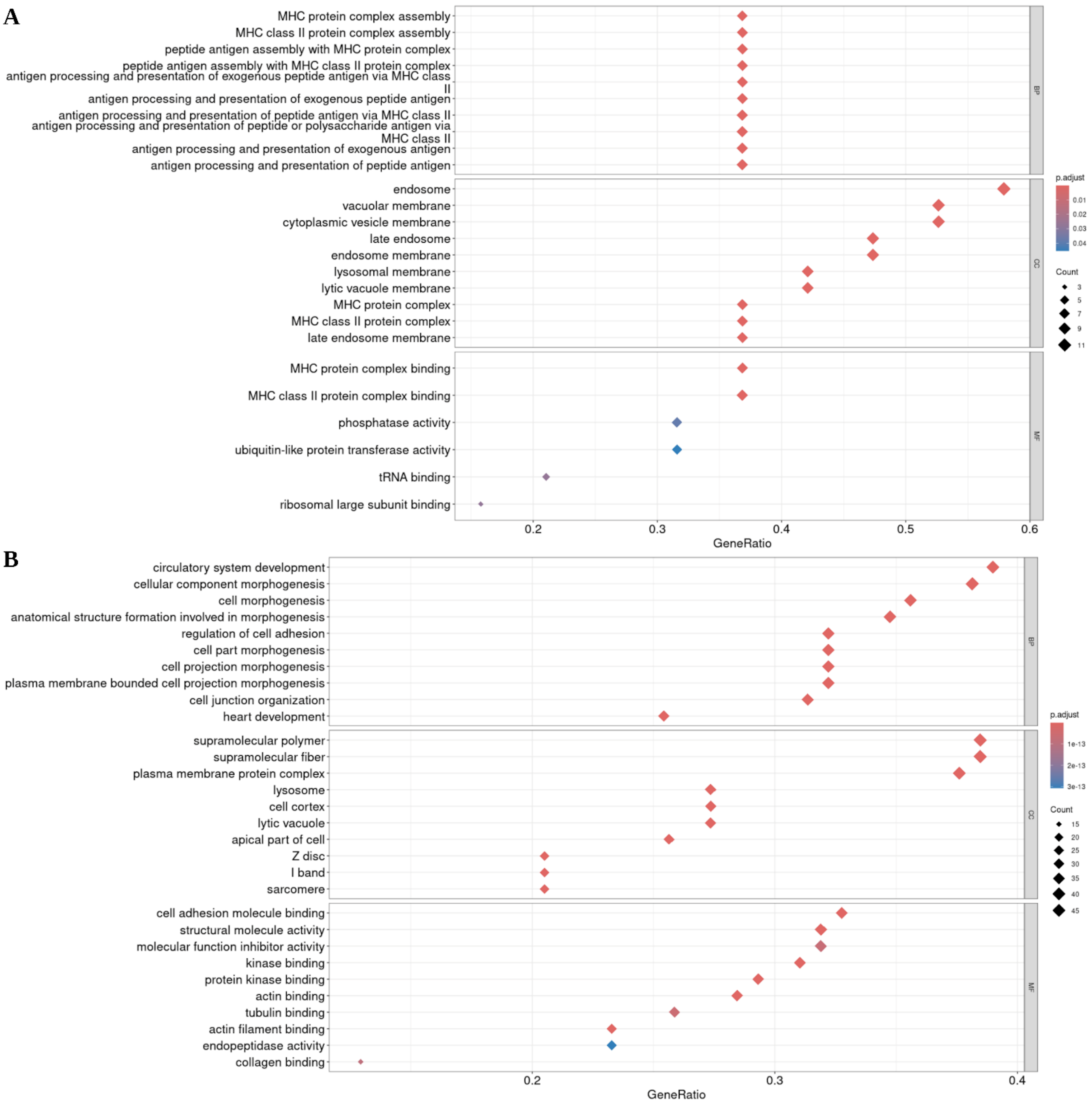

Supplementary Figure 3: Gene Ontology (GO) enrichment analysis of genes within core and cloud contigs. This figure presents the results of Gene Ontology (GO) enrichment analysis for genes located within **(A)** core contigs and **(B)** cloud contigs. Each diamond represents a significantly enriched GO term within the biological process (BP), cellular component (CC) and molecular function (MF) categories. Diamond size corresponds to the number of genes associated with that GO term, while the color represents the adjusted p-value for enrichment.

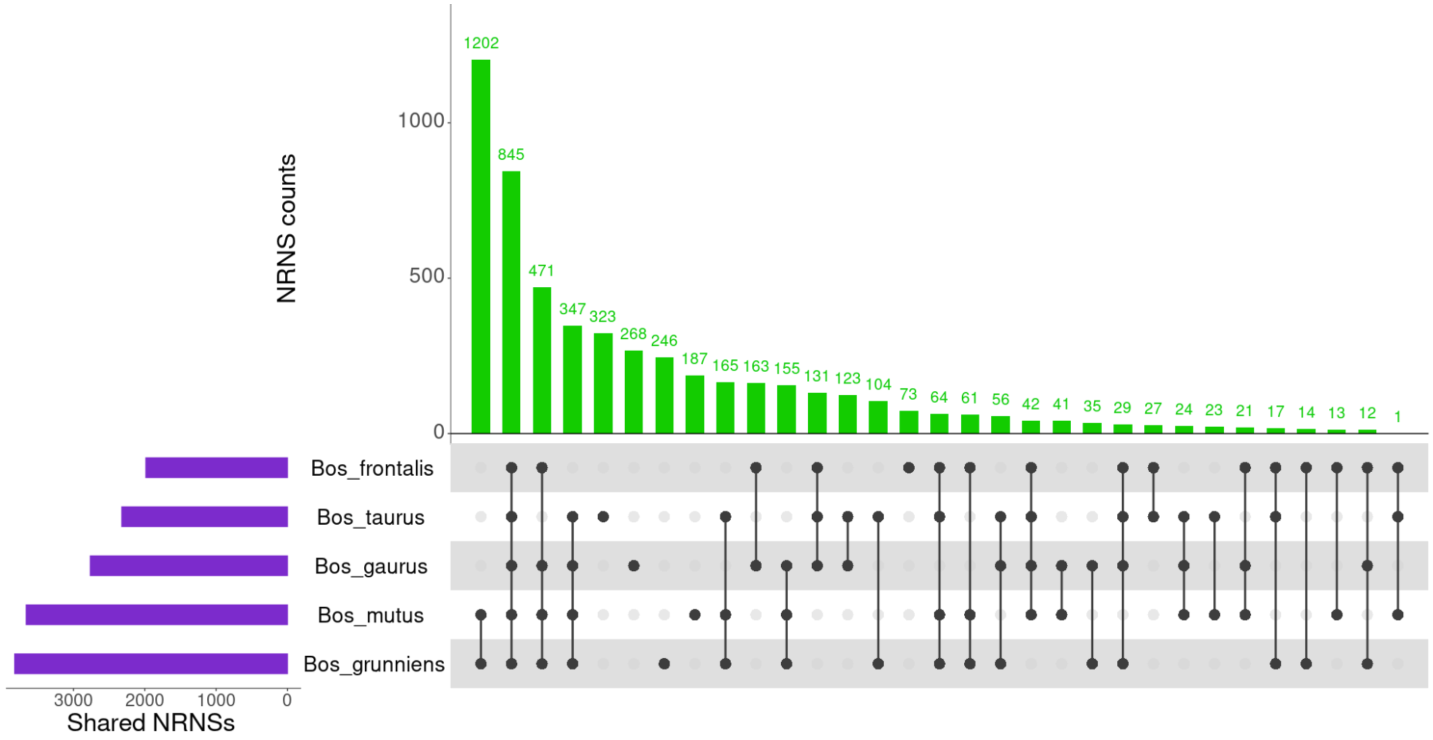

Supplementary Figure 4: Distribution of NRNS within the Bos genus. The upset plot depicts the distribution of NRNS across the Bos genus, highlighting the sharing of NRNS among different breeds.

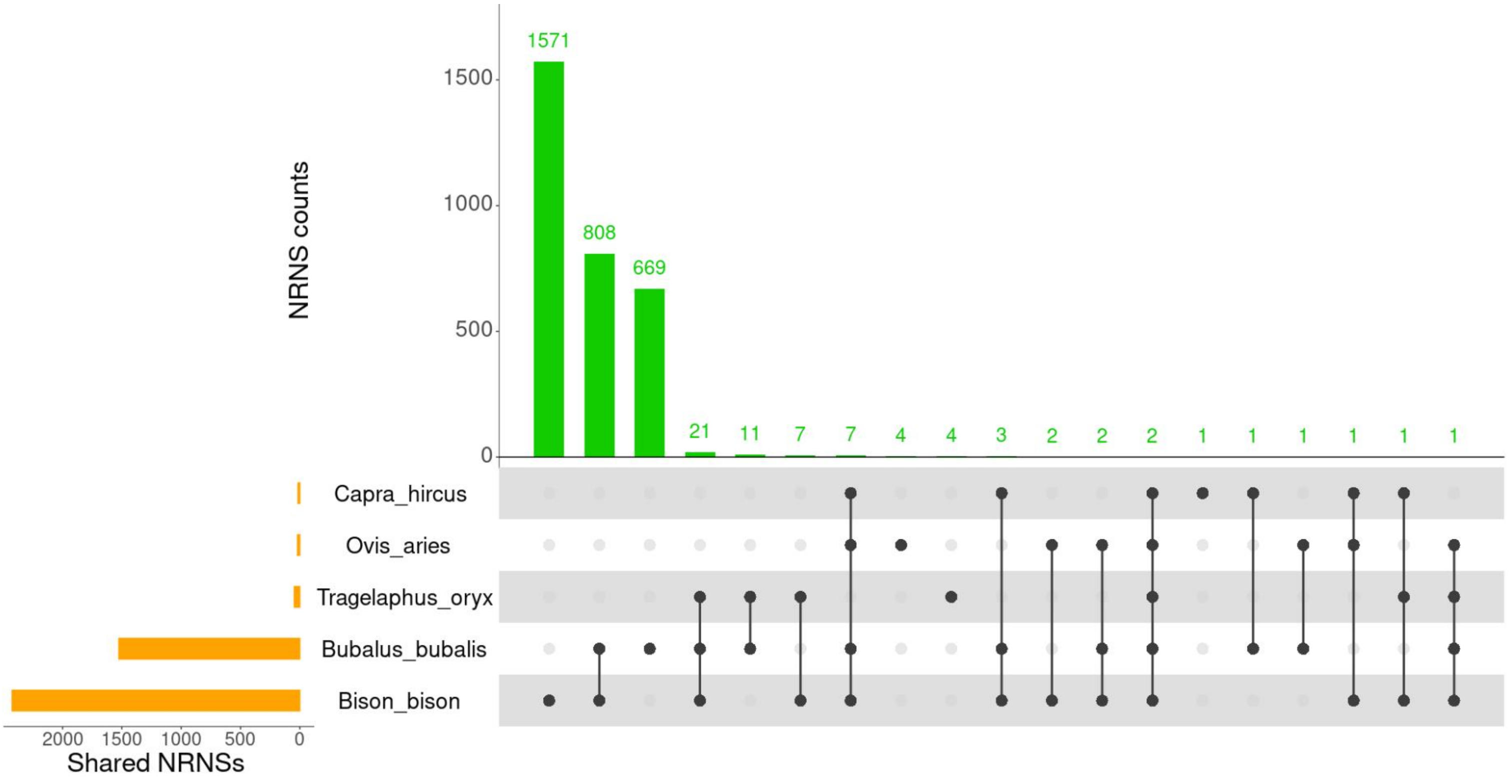

Supplementary Figure 5: Distribution of NRNS within the bovidae family. The upset plot depicts the distribution of NRNS across the bovidae family, highlighting the sharing of NRNS among different breeds.

Supplementary Table 1: Raw data quality assessment and filtering

Sample name	Before filtering				After filtering			
	Total reads (M)	Total bases (Gb)	Q20 bases (%)	GC content (%)	Total reads (M)	Total bases (Gb)	Q20 bases (%)	GC content (%)
Gir_02	1509.5	226.4	87.58	48.32	1016	138.2	96.64	47.13
Gir_04	771.9	115.8	95.39	47.94	676.86	93.24	98.2	47.51
Gir_06	753.9	113.1	95.06	48.17	651.24	89.65	98.1	47.74
Gir_08	864.2	129.6	95.15	47.07	749.28	102.7	98.1	46.58
Gir_09	832.1	124.8	96.14	47.52	750.17	103.5	98.33	47.23
Gir_10	747.1	112.1	95.52	45.75	659.18	91.03	98.12	45.42
Gir_11	994.5	149.2	95.87	45.56	890.07	123	98.11	45.21
Gir_12	912.4	136.9	96.73	46.52	835.66	115.2	98.61	46.26
Gir_250	742.9	112.2	97.2	42.3	707.6	104.0	98.3	42.0
Gir_376	605.2	91.4	96.8	42.1	565.3	78.4	98.3	40.9
Gir_383	459.6	69.4	96.7	43.8	430.6	61.3	98.2	43.1
Gir_390	506.3	76.5	97.0	43.9	481.5	66.9	98.3	43.1
Gir_446	540.8	81.7	96.9	42.7	503.3	68.5	98.4	41.0
Gir_713	483.9	73.1	96.7	43.7	455.8	63.4	98.1	42.7
Gir_885	468.7	70.8	97.3	43.2	440.3	60.1	98.7	41.7
Gir_890	503.2	76.0	96.8	44.0	475.3	65.1	98.2	42.7

Hariana_11	495.2	74.8	96.9	44.3	466.9	64.5	98.3	43.2
Hariana_16	633.1	95.6	96.8	42.6	597.4	88.7	98.0	42.5
Hariana_26	602.8	91.0	96.9	42.2	562.8	77.4	98.4	40.7
Hariana_27D	658.0	99.4	97.5	43.3	624.5	87.1	98.7	42.2
Hariana_27H	558.5	84.3	97.1	44.4	530.1	72.1	98.4	43.2
Hariana_29	489.6	73.9	96.5	42.1	459.0	63.3	98.3	40.7
Hariana_31	561.4	84.8	97.2	43.3	534.4	74.3	98.3	42.4
Hariana_39	513.1	77.5	97.0	44.1	484.2	67.2	98.2	43.1
Hariana_40	626.9	94.7	97.1	44.1	594.4	81.9	98.4	43.1
Kankrej_106	777.1	116.6	96.85	44.82	720.65	99.4	98.48	44.53
Kankrej_107	922.4	138.4	96.7	46.33	848.55	117.3	98.45	46.09
Kankrej_138	963.1	144.5	96.09	44.75	874.17	120.8	98.15	44.52
Kankrej_59	1046	156.9	96.03	46.96	947.58	129.3	98.1	46.64
Kankrej_60	970.3	145.6	96.21	46.27	877.34	118.2	98.43	45.87
Kankrej_61	739.4	110.9	96.82	46.61	681.48	92.54	98.63	46.28
Ladakhi_198	1066	159.9	96.48	44.45	977.2	135	98.46	44.21
Ladakhi_199	1117	167.5	96.59	44.81	1027.93	142.1	98.48	44.59
Red_Sindhi_133	988.4	148.3	94.91	45.24	858.35	118.4	97.69	44.74

Red_Sindhi_134	818.1	122.7	95.02	45.96	721.01	99.44	97.67	45.66
Red_Sindhi_135	766.7	115	95.08	45.22	670.15	92.56	97.72	44.77
Red_Sindhi_36	842.9	126.4	95.97	44.67	756.66	104.6	98.23	44.37
Red_Sindhi_37	706.5	106	95.51	44.83	629.02	86.88	98.05	44.5
Red_Sindhi_38	864	129.6	95.31	46.45	763.65	105.2	98.01	46.1
Sahiwal_21	773.5	116	95.64	44.87	688.96	95.09	98.07	44.49
Sahiwal_22	783.6	117.5	95.48	46.42	693.64	95.52	98.03	46.1
Sahiwal_23	812.8	121.9	95.2	44.84	708.06	97.67	98	44.48
Sahiwal_231	438.9	66.3	96.5	43.4	410.9	55.9	98.4	42.1
Sahiwal_24	665.3	99.79	95.12	45.08	577.84	79.69	97.98	44.78
Sahiwal_247	556.2	84.0	96.8	43.4	514.7	69.2	98.4	41.6
Sahiwal_26	691.5	103.7	94.72	46.7	593.62	81.77	97.79	46.4
Sahiwal_28	863.9	129.6	94.96	44.79	740.62	102.3	97.99	44.33
Sahiwal_402	574.6	86.8	97.1	42.9	545.2	78.8	98.3	42.5
Sahiwal_451	477.4	72.1	96.3	44.9	450.5	61.5	98.2	43.6
Sahiwal_605	554.2	83.7	96.7	43.9	521.8	73.2	98.2	43.0
Sahiwal_804	375.1	56.6	96.5	43.4	353.8	49.3	98.2	42.3
Sahiwal_808	516.2	77.9	96.9	44.1	487.1	68.4	98.2	43.2

Sahiwal_825	555.0	83.8	96.7	41.7	520.9	76.8	98.0	41.6
Sahiwal_857	568.1	85.8	96.6	44.8	538.3	73.9	98.3	43.6
Tharparkar_04	500.1	75.5	96.6	42.6	461.4	65.1	98.1	41.4
Tharparkar_05	503.6	76.0	96.8	44.0	473.6	66.6	98.3	43.0
Tharparkar_06	477.6	72.1	96.6	44.1	452.7	62.6	98.2	43.1
Tharparkar_07	574.0	86.7	96.6	44.4	542.8	75.3	98.2	43.3
Tharparkar_09D	870.4	131.4	97.0	42.9	815.3	113.4	98.5	41.7
Tharparkar_09H	449.7	67.9	97.0	43.4	426.2	59.6	98.2	42.4
Tharparkar_142	906.2	135.9	95.92	45.83	818.03	113	98.06	45.55
Tharparkar_143	835.7	125.4	95.71	44.23	747.49	103.3	98	43.99
Tharparkar_144	914.9	137.2	95.87	44.68	825.29	114	98.01	44.42
Tharparkar_145	907.2	136.1	95.93	45.64	818.28	113.1	98.07	45.39
Tharparkar_146	808.1	121.2	96.03	45.99	730.57	100.8	98.13	45.75
Tharparkar_17	512.7	77.4	96.0	41.9	471.7	66.8	98.1	40.6
Tharparkar_19	461.2	69.6	96.9	42.0	431.7	59.9	98.4	40.8
Tharparkar_56	999.5	149.9	96.82	46.24	928.76	126.5	98.51	45.94

Supplementary Table 2: Data statistics for read alignment against Brahman reference genome

Sample name	Total reads	Reads Aligned (PE)	Reads aligned (SE)	Reads unaligned (PE)	Reads unaligned (SE)	Overall alignment rate (%)
Gir_02	507997103	459762262	80744477	48234841	15725205	98.45
Gir_04	338428593	298780328	67268888	39648265	12027642	98.22
Gir_06	325622446	288839186	61976283	36783260	11590237	98.22
Gir_08	374637515	328087358	81765656	46550157	11334658	98.49
Gir_09	375086718	362285764	14582813	12800954	11019095	98.53
Gir_10	329590320	318847143	12690658	10743177	8795696	98.67
Gir_11	445034157	432492187	13558598	12541970	11525342	98.71
Gir_12	417830092	399370496	17382184	18459596	19537008	97.66
Gir_250	353817506	336988907	24798182	16828599	8859016	98.75
Gir_376	282673113	271725876	15974468	10947237	5920006	98.95
Gir_383	215292660	207531675	12061111	7760985	3460859	99.2
Gir_390	240735523	235026224	7494048	5709299	3924550	99.18
Gir_446	251642582	243433021	10573172	8209561	5845950	98.84
Gir_713	227919515	221206764	9475574	6712751	3949928	99.13
Gir_885	220141794	210011450	14671274	10130344	5589414	98.73

Gir_890	237669479	232108397	7045819	5561082	4076345	99.14
Hariana _11	233454640	226766434	9271915	6688206	4104497	99.12
Hariana_16	298723515	282361802	27105337	16361713	5618089	99.06
Hariana_26	281388635	272012880	13156391	9375755	5595119	99.01
Hariana_27D	312264906	296027049	24833657	16237857	7642057	98.78
Hariana_27H	265064510	259951855	5737662	5112655	4487648	99.15
Hariana_29	229513124	221133124	11889349	8380000	4870651	98.94
Hariana_31	267212810	259240523	10702147	7972287	5242427	99.02
Hariana_39	242101330	235424928	9005896	6676402	4346908	99.1
Hariana_40	297191258	290033100	9753233	7158158	4563083	99.23
Kankrej_106	360325369	346832114	17192809	13493255	9793701	98.64
Kankrej_107	424276562	410840121	16826294	13436441	10046588	98.82
Kankrej_138	437086873	420159487	20349582	16927386	13505190	98.46
Kankrej_59	473789789	442775406	44833626	31014383	17195140	98.19
Kankrej_60	438667698	407578803	49717564	31088895	12460226	98.58
Kankrej_61	340739127	318631854	32719463	22107273	11495083	98.31
Ladakhi_198	488601127	469337229	23853313	19263898	14674483	98.5
Ladakhi_199	513962967	486964688	35115531	26998279	18881027	98.16

Red Sindhi_133	429175597	407843812	28129227	21331785	14534343	98.31
Red Sindhi_134	360507191	344816950	19502408	15690241	11878074	98.35
Red Sindhi_135	335077202	320702088	17643420	14375114	11106808	98.34
Red Sindhi_36	378328038	349788095	25767027	28539943	31312859	95.86
Red Sindhi_37	314511478	300026258	20749497	14485220	8220943	98.69
Red Sindhi_38	381823416	358783821	32840300	23039595	13238890	98.27
Sahiwal_21	344478549	331491980	16754066	12986569	9219072	98.66
Sahiwal_22	346821436	327452948	26435844	19368488	12301132	98.23
Sahiwal_23	354027668	339446110	18783542	14581558	10379574	98.53
Sahiwal_231	205460222	198572839	8959294	6887383	4815472	98.83
Sahiwal_24	288920840	278672214	12564051	10248626	7933201	98.63
Sahiwal_247	257356611	248836088	10843922	8520523	6197124	98.8
Sahiwal_26	296810854	274982601	29452336	21828253	14204170	97.61
Sahiwal_27	370311024	355644524	17349825	14666500	11983175	98.38
Sahiwal_402	272602806	257171014	20445044	15431792	10418540	98.09
Sahiwal_451	225272547	220438352	5617083	4834195	4051307	99.1
Sahiwal_605	260878160	251615145	13914471	9263015	4611559	99.12
Sahiwal_804	176885776	171564027	7611756	5321749	3031742	99.14

Sahiwal_808	243572022	234007142	13812757	9564880	5317003	98.91
Sahiwal_825	260467706	243651132	24525046	16816574	9108102	98.25
Sahiwal_857	269151514	263290084	7607176	5861430	4115684	99.24
Tharparkar_04	230689529	216928878	22550160	13760651	4971142	98.92
Tharparkar_05	236807535	227351965	14666732	9455570	4244408	99.1
Tharparkar_06	226346393	220833141	7173393	5513252	3853111	99.15
Tharparkar_07	271424640	264156588	10207675	7268052	4328429	99.2
Tharparkar_09D	407647013	387836991	29156587	19810022	10463457	98.72
Tharparkar_09H	213117540	206395461	9935084	6722079	3509074	99.18
Tharparkar_142	409014416	390479943	22692582	18534473	14376364	98.24
Tharparkar_143	373745984	357254462	20584978	16491522	12398066	98.34
Tharparkar_144	412647485	392857021	23375039	19790464	16205889	98.04
Tharparkar_145	409138428	388650820	25562801	20487608	15412415	98.12
Tharparkar_146	365286841	343309634	24393102	21977207	19561312	97.32
Tharparkar_17	235829546	220931519	24266214	14898027	5529840	98.83
Tharparkar_19	215859955	207326934	12465313	8533021	4600729	98.93
Tharparkar_56	464380004	425157399	53901546	39222605	24543664	97.36

Supplementary Table 3: Summary statistics of NRNS-filtered contigs

Sample_name	Total Contigs	Contig after filter >1kb	Removed sequence	N50	L50	Total length (bp)	Total sequence
Gir_02	22443	5087	2102	3571	577	8258954	2985
Gir_04	21102	4753	2076	3084	556	6961887	2677
Gir_06	18456	4080	1112	2860	654	7277116	2968
Gir_08	21188	4326	1187	3378	638	8551045	3139
Gir_09	18050	3741	760	3191	623	7932455	2981
Gir_10	19094	3702	1108	4047	506	7831697	2594
Gir_11	17439	3466	799	4196	491	8224716	2667
Gir_12	26249	6249	834	3115	1148	14145882	5415
Gir_250	18580	3229	350	3784	572	8336926	2879
Gir_376	13182	3038	283	2993	669	6936350	2755
Gir_383	11430	2681	231	3514	534	6781689	2450
Gir_390	11594	2716	241	3404	540	6714761	2475
Gir_446	14993	3094	269	2712	729	6606766	2825
Gir_713	12164	2818	221	3061	591	6615935	2597
Gir_885	12453	2741	206	2708	634	5965994	2535

Gir_890	12556	2696	157	3124	579	6587084	2539
Hariana_11	13696	2862	327	3345	3345	6781981	2535
Hariana_16	15672	3030	353	3996	504	8001153	2677
Hariana_26	13643	3104	175	2635	749	6831211	2929
Hariana_27D	16181	3290	344	3267	654	7879964	2946
Hariana_27H	12830	2857	234	3136	594	6790206	2623
Hariana_29	12615	2827	181	2535	697	6060325	2646
Hariana_31	13515	3019	323	3407	587	7384445	2696
Hariana_39	13927	2803	274	3402	540	6909479	2529
Hariana_40	12371	2636	230	3783	493	6957694	2406
Kankrej_106	15923	3420	683	4389	489	8631991	2737
Kankrej_107	16034	3509	691	3842	534	8223365	2818
Kankrej_138	24009	5735	2839	4310	527	8951546	2896
Kankrej_59	19727	3817	841	3934	557	8908307	2976
Kankrej_60	17590	3515	718	3901	512	8172443	2797
Kankrej_61	18001	3686	708	3422	589	8033151	2978
Ladakhi_198	19520	4181	1057	4491	537	9894247	3124
Ladakhi_199	20675	3922	624	4273	583	10184496	3298

Red Sindhi_133	18737	4014	830	4077	572	9476636	3184
Red Sindhi_134	17390	3953	893	4054	576	9270652	3060
Red Sindhi_135	16759	3605	723	3887	543	8420858	2882
Red Sindhi_36	18251	4310	1311	4111	523	9064418	2999
Red Sindhi_37	22345	5405	2649	4520	505	8767863	2756
Red Sindhi_38	17426	3849	917	4124	527	8826676	2932
Sahiwal_21	15184	3487	490	4265	533	9157136	2997
Sahiwal_22	16650	3631	638	3921	562	8751648	2993
Sahiwal_23	17539	3632	714	4197	520	8948821	2918
Sahiwal_231	12433	2709	140	2618	666	5894309	2569
Sahiwal_24	15297	3533	469	3751	576	8637049	3064
Sahiwal_247	15478	2988	214	2782	695	6650350	2774
Sahiwal_26	16348	3512	544	3363	637	7950387	2968
Sahiwal_27	21033	4207	1381	4408	518	8913680	2826
Sahiwal_402	21642	3014	239	3118	656	7178179	2775
Sahiwal_451	12698	2762	237	3057	595	6419128	2525
Sahiwal_605	16560	3072	594	3696	490	7165829	2478
Sahiwal_804	11539	2576	174	2768	616	5704745	2402

Sahiwal_808	20540	4994	2526	3573	519	6975851	2468
Sahiwal_825	17931	3115	336	3338	631	7563891	2779
Sahiwal_857	11691	2610	200	3481	494	6717120	2410
Tharparkar_04	12303	2851	277	3056	621	6565746	2574
Tharparkar_05	13868	2711	346	3700	477	6829291	2365
Tharparkar_06	10798	2666	197	3299	568	6618074	2469
Tharparkar_07	11676	2745	254	3537	525	7019634	2491
Tharparkar_09D	23001	3485	436	3426	676	8341073	3049
Tharparkar_09H	11637	2729	214	3119	597	6400534	2515
Tharparkar_142	24024	5340	2274	3651	607	8638627	3066
Tharparkar_143	16667	3594	615	4070	550	9157859	2979
Tharparkar_144	26592	6067	3101	4388	526	9308177	2966
Tharparkar_145	21925	4014	1002	3802	564	8758390	3012
Tharparkar_146	24266	5964	762	3195	1051	13556845	5202
Tharparkar_17	13813	2973	230	2708	710	6485480	2743
Tharparkar_19	12565	2643	130	2624	650	5794527	2513
Tharparkar_56	28616	4965	1634	3759	628	9536970	3331

Supplementary Table 4: Status of contaminants

Contaminants	Counts
environmental samples	30881
Theileria annulata	12417
Theileria orientalis	3954
Babesia bigemina	614
Anaplasma marginale	499
Clostridium botulinum	423
Others	1441

Note: The "others" category primarily includes apicomplexans.

Supplementary Table 5: Gene annotation of NRNSs

Tools	Total transcripts	Hit to bovidae family	Hit to chordata NR database	Non-redundant genes	Combined non-redundant genes
Augustus	1856	699	64	376	880
String-tie	7991	3631	432	634	
**Placement**	**NRNS**	**NRNS in genes**		**Non-redundant genes**	**Combined non-redundant genes**
One end placed	1766	764		510	679
Two end placed	536	251		224	
**Final Combined non-redundant genes**					**1453**

Supplementary Table 6: Repeat annotation of NRNS

**sequences**	13065
**Total length**	40973925 bp (40973925 bp excl N/X-runs)
**GC level**	42.84 %
**bases masked**	17527709 bp (42.78 %)

**Repeat Category**	**Number of elements***	**Length occupied**	**Percentage of sequence**
**SINEs:**	28687	3952615 bp	9.65 %
**Alu/B1**	0	0 bp	0 %
**MIRs**	2653	347905 bp	0.85 %
**LINEs:**	27911	9640410 bp	23.53 %
**LINE1**	13795	5595521 bp	13.66 %
**LINE2**	1810	396122 bp	0.97 %
**L3/CR1**	215	40966 bp	0.10 %
**RTE**	12088	3607608 bp	8.80 %

**DNA elements:**	3204	641962 bp	1.57 %
**hAT-Charlie**	1807	346503 bp	0.85 %
**TcMar-Tigger**	651	161607 bp	0.39 %
**Unclassified:**	23	4019 bp	0.01 %
**Total interspersed repeats:**		16150180 bp	39.42 %
**Small RNA:**	3779	622177 bp	1.52 %
**Satellites:**	1127	1346506 bp	3.29 %

Supplementary Table 7: DNA-seq samples accession included in study

Sample Name	Study_ID_INDA	INDA sample accession	INSDC accession
Gir_02	INRP000053	INS0003369	ERS15934556
Gir_04	INRP000053	INS0003352	ERS15933514
Gir_06	INRP000053	INS0003354	ERS15935106
Gir_08	INRP000053	INS0003375	ERS15933523
Gir_09	INRP000053	INS0003381	ERS15933710
Gir_10	INRP000053	INS0003356	ERS15935115
Gir_11	INRP000053	INS0003357	ERS15934638
Gir_12	INRP000053	INS0003402	ERS15933747
Gir_250	INRP000159	INS0004914	ERS20864995
Gir_376	INRP000159	INS0004918	ERS20865408
Gir_383	INRP000159	INS0004894	ERS20842157
Gir_390	INRP000159	INS0004895	ERS20847879
Gir_446	INRP000159	INS0004920	ERS20865411
Gir_713	INRP000159	INS0004902	ERS20848420
Gir_885	INRP000159	INS0004923	ERS20880959
Gir_890	INRP000159	INS0004907	ERS20864988

Hariana _11	INRP000159	INS0004891	ERS20820295
Hariana_16	INRP000159	INS0004909	ERS20864990
Hariana_26	INRP000159	INS0004915	ERS15934366
Hariana_27D	INRP000159	INS0004916	ERS20864996
Hariana_27H	INRP000159	INS0004892	ERS20820296
Hariana_29	INRP000159	INS0004917	ERS20864997
Hariana_31	INRP000159	INS0004893	ERS20842156
Hariana_39	INRP000159	INS0004896	ERS20847888
Hariana_40	INRP000159	INS0004897	ERS20848091
Kankrej_106	INRP000053	INS0003453	ERS15936274
Kankrej_107	INRP000053	INS0003454	ERS15936279
Kankrej_138	INRP000053	INS0003472	ERS15936278
Kankrej_59	INRP000053	INS0003372	ERS15935283
Kankrej_60	INRP000053	INS0003462	ERS15935842
Kankrej_61	INRP000053	INS0003422	ERS15934368
Ladakhi_198	INRP000053	INS0003382	ERS15935122
Ladakhi_199	INRP000053	INS0003383	ERS15935101
Red Sindhi_133	INRP000053	INS0003483	ERS15947722

Red Sindhi_134	INRP000053	INS0000661	ERS15960199
Red Sindhi_135	INRP000053	INS0003484	ERS15947734
Red Sindhi_36	INRP000053	INS0003411	ERS15935087
Red Sindhi_37	INRP000053	INS0003412	ERS15935287
Red Sindhi_38	INRP000053	INS0003413	ERS15934557
Sahiwal_21	INRP000053	INS0003361	ERS15935281
Sahiwal_22	INRP000053	INS0003362	ERS15934588
Sahiwal_23	INRP000053	INS0003363	ERS15933518
Sahiwal_231	INRP000159	INS0004912	ERS20864993
Sahiwal_24	INRP000053	INS0003364	ERS15935093
Sahiwal_247	INRP000159	INS0004913	ERS20864994
Sahiwal_26	INRP000053	INS0003365	ERS15933519
Sahiwal_27	INRP000053	INS0003367	ERS15935128
Sahiwal_402	INRP000159	INS0004919	ERS20865410
Sahiwal_451	INRP000159	INS0004898	ERS20848402
Sahiwal_605	INRP000159	INS0004900	ERS20848418
Sahiwal_804	INRP000159	INS0004904	ERS20848422
Sahiwal_808	INRP000159	INS0004905	ERS20864976

Sahiwal_825	INRP000159	INS0004922	ERS20878324
Sahiwal_857	INRP000159	INS0004906	ERS20864987
Tharparkar_04	INRP000159	INS0004921	ERS20865412
Tharparkar_05	INRP000159	INS0004899	ERS20848403
Tharparkar_06	INRP000159	INS0004901	ERS20848419
Tharparkar_07	INRP000159	INS0004903	ERS20848421
Tharparkar_09D	INRP000159	INS0004924	ERS20880960
Tharparkar_09H	INRP000159	INS0004908	ERS20864989
Tharparkar_142	INRP000053	INS0003475	ERS15936288
Tharparkar_143	INRP000053	INS0003476	ERS15936291
Tharparkar_144	INRP000053	INS0003477	ERS15936293
Tharparkar_145	INRP000053	INS0003463	ERS15936281
Tharparkar_146	INRP000053	INS0003464	ERS15936283
Tharparkar_17	INRP000159	INS0004910	ERS20864991
Tharparkar_19	INRP000159	INS0004911	ERS20864992
Tharparkar_56	INRP000053	INS0003433	ERS15935286

Supplementary Table 8: RNA-seq samples accession included in study

Study_ID_INDA	INDA sample accession	INSDC accession
INRP000128	INS0004350	ERS18084896
INRP000128	INS0004351	ERS18084984
INRP000128	INS0004352	ERS18084945
INRP000128	INS0004353	ERS18083834
INRP000128	INS0004354	ERS18084991
INRP000128	INS0004355	ERS18083836
INRP000128	INS0004356	ERS18084713
INRP000128	INS0004357	ERS18083837
INRP000128	INS0004358	ERS18084028
INRP000128	INS0004359	ERS18271704
INRP000128	INS0004360	ERS18084941
INRP000128	INS0004361	ERS18084721
INRP000128	INS0004362	ERS18083844
INRP000128	INS0004363	ERS18084985
INRP000128	INS0004364	ERS18084986

INRP000128	INS0004349	ERS18083833
INRP000128	INS0004365	ERS18083904
INRP000128	INS0004366	ERS18084936
INRP000128	INS0004367	ERS18084579
INRP000128	INS0004368	ERS18084975
INRP000128	INS0004369	ERS18084938
INRP000128	INS0004370	ERS18084707
INRP000128	INS0004371	ERS18084082
INRP000128	INS0004372	ERS18084085
INRP000128	INS0004373	ERS18084637
INRP000128	INS0004374	ERS18084982
INRP000128	INS0004375	ERS18084183
INRP000128	INS0004376	ERS18084937
INRP000128	INS0004377	ERS18084405
INRP000128	INS0004378	ERS18084409
INRP000128	INS0004379	ERS18084709
INRP000128	INS0004380	ERS18084987
INRP000128	INS0004381	ERS18084702

INRP000128	INS0004382	ERS18084410
INRP000128	INS0004383	ERS18084973
INRP000128	INS0004384	ERS18084944
INRP000128	INS0004385	ERS18084421
INRP000128	INS0004386	ERS18084940
INRP000128	INS0004387	ERS18271703
INRP000128	INS0004388	ERS18084550
INRP000128	INS0004389	ERS18084988
INRP000128	INS0004390	ERS18084573
INRP000128	INS0004391	ERS18084572
INRP000128	INS0004392	ERS18084974
INRP000128	INS0004393	ERS18084578
INRP000128	INS0004394	ERS18077802
INRP000128	INS0004395	ERS18084577
