# Supplementary material for: Constructing a draft Indian cattle pangenome using short-read sequencing
Source: Commun Biol. 2025 Apr 13;8:605. doi: 10.1038/s42003-025-07978-0 (PMC11994783; doi:10.1038/s42003-025-07978-0)
Supplement: Supplementary file 6 — Supplementary Data 5 [file 42003_2025_7978_MOESM6_ESM.docx]

| **Sample_ID** | **Reference** | | | | | **Pan_genome** | | | | | **Panbase** | | | | |
| --- | --- | --- | --- | --- | --- | --- | --- | --- | --- | --- | --- | --- | --- | --- | --- |
|  | **Total reads** | **Mapped reads** | **Mapped (%)** | **Properly paired** | **properly paired (%)** | **Total reads** | **Mapped reads** | **Mapped (%)** | **Properly paired** | **Properly paired (%)** | **Total reads** | **Mapped reads** | **Mapped (%)** | **Properly paired** | **Properly paired (%)** |
| LGP-100 | 842798048 | 825833122 | 97.99 | 791671754 | 93.93 | 842798048 | 834400171 | 99 | 800201462 | 94.95 | 842798048 | 822220811 | 97.56 | 789541006 | 93.68 |
| LGP-112 | 492526544 | 479069419 | 97.27 | 416727572 | 84.61 | 492526544 | 484702643 | 98.41 | 421813940 | 85.64 | 492526544 | 475974719 | 96.64 | 415021804 | 84.26 |
| LGP-115 | 872627988 | 848886911 | 97.28 | 789797212 | 90.51 | 872627988 | 858833214 | 98.42 | 799259118 | 91.59 | 872627988 | 843544769 | 96.67 | 786587606 | 90.14 |
| LGP-117 | 957439460 | 937182671 | 97.88 | 893555750 | 93.33 | 957439460 | 947081872 | 98.92 | 903321148 | 94.35 | 957439460 | 933029641 | 97.45 | 891164606 | 93.08 |
| LGP-118 | 900829972 | 878682427 | 97.54 | 845432524 | 93.85 | 900829972 | 888525550 | 98.63 | 855313902 | 94.95 | 900829972 | 873666486 | 96.98 | 842406874 | 93.51 |
| LGP-120 | 717958376 | 700649213 | 97.59 | 548352570 | 76.38 | 717958376 | 707930335 | 98.6 | 554257524 | 77.2 | 717958376 | 696307198 | 96.98 | 546056394 | 76.06 |
| LGP-123 | 798431868 | 777408160 | 97.37 | 732433430 | 91.73 | 798431868 | 786248580 | 98.47 | 741020148 | 92.81 | 798431868 | 772573469 | 96.76 | 729549174 | 91.37 |
| LGP-129 | 788189558 | 770569177 | 97.76 | 733035086 | 93 | 788189558 | 778267056 | 98.74 | 740711460 | 93.98 | 788189558 | 767304247 | 97.35 | 731268918 | 92.78 |
| LGP-131 | 764272758 | 747731694 | 97.84 | 715058680 | 93.56 | 764272758 | 755230179 | 98.82 | 722634778 | 94.55 | 764272758 | 744485499 | 97.41 | 713233118 | 93.32 |
| LGP-147 | 962762652 | 932781533 | 96.89 | 873864486 | 90.77 | 962762652 | 942451975 | 97.89 | 883262084 | 91.74 | 962762652 | 928585399 | 96.45 | 871649198 | 90.54 |
| LGP-148 | 709526598 | 689444804 | 97.17 | 650300578 | 91.65 | 709526598 | 696841007 | 98.21 | 657568016 | 92.68 | 709526598 | 686002328 | 96.68 | 648341588 | 91.38 |
| LGP-150 | 939913700 | 920036013 | 97.89 | 866910906 | 92.23 | 939913700 | 923078520 | 98.21 | 869874830 | 92.55 | 939913700 | 917298357 | 97.59 | 865363746 | 92.07 |
| LGP-151 | 802291606 | 781611850 | 97.42 | 743695510 | 92.7 | 802291606 | 789486488 | 98.4 | 751530756 | 93.67 | 802291606 | 778209219 | 97.00 | 741764790 | 92.46 |
| LGP-163 | 769581352 | 751123772 | 97.6 | 662027256 | 86.02 | 769581352 | 758589542 | 98.57 | 668783610 | 86.9 | 769581352 | 746539271 | 97.01 | 659379450 | 85.68 |
| LGP174 | 824653642 | 805923206 | 97.73 | 780761736 | 94.68 | 824653642 | 814990438 | 98.83 | 789944084 | 95.79 | 824653642 | 801999853 | 97.25 | 778336438 | 94.38 |
| LGP176 | 923948922 | 903369490 | 97.77 | 882555086 | 95.52 | 923948922 | 912709485 | 98.78 | 892096554 | 96.55 | 923948922 | 899170164 | 97.32 | 879840700 | 95.23 |
| LGP183 | 800185794 | 780463042 | 97.54 | 751353158 | 93.9 | 800185794 | 789407457 | 98.65 | 760340274 | 95.02 | 800185794 | 775698697 | 96.94 | 748190862 | 93.50 |
| LGP190 | 981160568 | 960588149 | 97.9 | 912499264 | 93 | 981160568 | 970810760 | 98.95 | 922645626 | 94.04 | 981160568 | 955473505 | 97.38 | 909207370 | 92.67 |
| LGP191 | 826597446 | 810463083 | 98.05 | 773233764 | 93.54 | 826597446 | 818743447 | 99.05 | 781484098 | 94.54 | 826597446 | 807035926 | 97.63 | 771277646 | 93.31 |
| LGP194 | 785223930 | 763218201 | 97.2 | 702589442 | 89.48 | 785223930 | 771932806 | 98.31 | 710990482 | 90.55 | 785223930 | 758357352 | 96.58 | 699584538 | 89.09 |
| LGP196 | 754079742 | 735855842 | 97.58 | 685644120 | 90.92 | 754079742 | 743927609 | 98.65 | 693530998 | 91.97 | 754079742 | 731825167 | 97.05 | 683104546 | 90.59 |
| LGP25 | 682081082 | 666674481 | 97.74 | 634641058 | 93.04 | 682081082 | 673567928 | 98.75 | 641461436 | 94.04 | 682081082 | 663407030 | 97.26 | 632766928 | 92.77 |
| LGP32 | 828170872 | 808193104 | 97.59 | 743570028 | 89.78 | 828170872 | 816813362 | 98.63 | 751749770 | 90.77 | 828170872 | 804458197 | 97.14 | 741525430 | 89.54 |
| LGP35 | 978092158 | 958917012 | 98.04 | 906812198 | 92.71 | 978092158 | 968864608 | 99.06 | 916614972 | 93.71 | 978092158 | 955716494 | 97.71 | 905241412 | 92.55 |
| LGP40 | 711310850 | 696937772 | 97.98 | 672562618 | 94.55 | 711310850 | 703774933 | 98.94 | 679369350 | 95.51 | 711310850 | 693878978 | 97.55 | 670782452 | 94.30 |
| LGP70 | 840975624 | 820370277 | 97.55 | 783550268 | 93.17 | 840975624 | 829299143 | 98.61 | 792347326 | 94.22 | 840975624 | 814784889 | 96.89 | 779914924 | 92.74 |
| LGP76 | 739377910 | 721107385 | 97.53 | 685156484 | 92.67 | 739377910 | 728962099 | 98.59 | 692864648 | 93.71 | 739377910 | 717648850 | 97.06 | 683266318 | 92.41 |
| LGP84 | 781578938 | 759426323 | 97.17 | 720381300 | 92.17 | 781578938 | 768236484 | 98.29 | 728970244 | 93.27 | 781578938 | 754520606 | 96.54 | 717466212 | 91.80 |
| LGP91 | 673048784 | 661019373 | 98.21 | 619978930 | 92.12 | 673048784 | 663277518 | 98.55 | 622061590 | 92.42 | 673048784 | 658406293 | 97.82 | 618294650 | 91.86 |
| LGP93 | 736381642 | 720186313 | 97.8 | 666538482 | 90.52 | 736381642 | 727747089 | 98.83 | 673808564 | 91.5 | 736381642 | 716080084 | 97.24 | 664019542 | 90.17 |
