# Supplementary material for: Constructing a draft Indian cattle pangenome using short-read sequencing
Source: Commun Biol. 2025 Apr 13;8:605. doi: 10.1038/s42003-025-07978-0 (PMC11994783; doi:10.1038/s42003-025-07978-0)
Supplement: Supplementary file 7 — Description of supplementary data [file 42003_2025_7978_MOESM7_ESM.docx]

**Description of Additional Supplementary Files:

File Name:** Supplementary Data 1

**Description:** NRNS sequences in FASTA format

**File Name:** Supplementary Data 2

**Description:** NRNS placed end VCF file

**File Name:** Supplementary Data 3

**Description:** Positions of NRNS with both ends placed on chromosomes

**File Name:** Supplementary Data 4

**Description:** Positions of NRNS with one end placed on chromosomes

**File Name:** Supplementary Data 5

**Description:** Short read mapping statistics for Brahman, pangenome, and PanBase
