# Supplementary material for: Constructing a draft Indian cattle pangenome using short-read sequencing
Source: Commun Biol. 2025 Apr 13;8:605. doi: 10.1038/s42003-025-07978-0 (PMC11994783; doi:10.1038/s42003-025-07978-0)
Supplement: Supplementary file 8 — Reporting summary [file 42003_2025_7978_MOESM8_ESM.pdf]

nature portfolio

Corresponding author(s): Benjamin D Rosen, Subeer S Majumdar

Last updated by author(s): Feb 28, 2025

Reporting Summary

Nature Portfolio wishes to improve the reproducibility of the work that we publish. This form provides structure for consistency and transparency in reporting. For further information on Nature Portfolio policies, see our Editorial Policies and the Editorial Policy Checklist.

Statistics

For all statistical analyses, confirm that the following items are present in the figure legend, table legend, main text, or Methods section.

n/a	Confirmed	
☐	☒	The exact sample size (*n*) for each experimental group/condition, given as a discrete number and unit of measurement
☒	☐	A statement on whether measurements were taken from distinct samples or whether the same sample was measured repeatedly
☒	☐	The statistical test(s) used AND whether they are one- or two-sided<br>*Only common tests should be described solely by name; describe more complex techniques in the Methods section.*
☒	☐	A description of all covariates tested
☒	☐	A description of any assumptions or corrections, such as tests of normality and adjustment for multiple comparisons
☒	☐	A full description of the statistical parameters including central tendency (e.g. means) or other basic estimates (e.g. regression coefficient) AND variation (e.g. standard deviation) or associated estimates of uncertainty (e.g. confidence intervals)
☒	☐	For null hypothesis testing, the test statistic (e.g. *F*, *t*, *r*) with confidence intervals, effect sizes, degrees of freedom and *P* value noted<br>*Give P values as exact values whenever suitable.*
☒	☐	For Bayesian analysis, information on the choice of priors and Markov chain Monte Carlo settings
☒	☐	For hierarchical and complex designs, identification of the appropriate level for tests and full reporting of outcomes
☒	☐	Estimates of effect sizes (e.g. Cohen's *d*, Pearson's *r*), indicating how they were calculated

Our web collection on statistics for biologists contains articles on many of the points above.

Software and code

Policy information about availability of computer code

Data collection	No software was used to collect the data
Data analysis	fastp v0.23.3<br>Bowtie2 v2.3.5.1<br>Samtools v1.2<br>MaSuRCA v4.0.3<br>CD-HIT v4.8.1<br>BLAST v2.9.0+<br>Popins2 v0.13.0<br>Augustus v3.4.0<br>STAR aligner v2.7.10<br>Stringtie v2.1.1<br>clusterProfiler<br>RepeatMasker v4.1.0<br>Picard Tools v3.1.1<br>GATK v4.3.0.0<br>PanGA pipeline<br>Fasta2Lineage

For manuscripts utilizing custom algorithms or software that are central to the research but not yet described in published literature, software must be made available to editors and reviewers. We strongly encourage code deposition in a community repository (e.g. GitHub). See the Nature Portfolio guidelines for submitting code & software for further information.

Data

Policy information about availability of data

All manuscripts must include a data availability statement. This statement should provide the following information, where applicable:

- Accession codes, unique identifiers, or web links for publicly available datasets
- A description of any restrictions on data availability
- For clinical datasets or third party data, please ensure that the statement adheres to our policy

The short-read sequencing dataset generated from the Illumina platform and used in this study has been submitted to the Indian Biological Data Centre (IBDC). The accession numbers for these datasets are detailed in Supplementary Table 7. Similarly, the accession numbers for the RNA-seq data used in the study are provided in Supplementary Table 8. All the data can also be accessed from the NCBI.

Research involving human participants, their data, or biological material

Policy information about studies with human participants or human data. See also policy information about sex, gender (identity/presentation), and sexual orientation and race, ethnicity and racism.

Reporting on sex and gender	No human participants, their data, or biological material was used
Reporting on race, ethnicity, or other socially relevant groupings	No human participants, their data, or biological material was used
Population characteristics	No human participants, their data, or biological material was used
Recruitment	No human participants, their data, or biological material was used
Ethics oversight	No human participants, their data, or biological material was used

Note that full information on the approval of the study protocol must also be provided in the manuscript.

Field-specific reporting

Please select the one below that is the best fit for your research. If you are not sure, read the appropriate sections before making your selection.

☒ Life sciences ☐ Behavioural & social sciences ☐ Ecological, evolutionary & environmental sciences

For a reference copy of the document with all sections, see nature.com/documents/nr-reporting-summary-flat.pdf

Life sciences study design

All studies must disclose on these points even when the disclosure is negative.

Sample size	The sample set included 16 individuals from the Gir breed, 15 from Sahiwal, 14 from Tharparkar, 9 from Hariana, 6 from Red Sindhi, 6 from Kankrej, and 2 from Ladakhi breeds, with all individuals being female. Samples were collected from either their native tract or farms maintaining the pure bred.
Data exclusions	All data were included and subsequently filtered based on the criteria described in the Methods section.
Replication	Data analysis is reproducible when using the same pipeline and parameters as described in Methods.
Randomization	Not relevant
Blinding	Not relevant

Reporting for specific materials, systems and methods

We require information from authors about some types of materials, experimental systems and methods used in many studies. Here, indicate whether each material, system or method listed is relevant to your study. If you are not sure if a list item applies to your research, read the appropriate section before selecting a response.

Materials & experimental systems

n/a	Involved in the study
☒	☐ Antibodies
☒	☐ Eukaryotic cell lines
☒	☐ Palaeontology and archaeology
☐	☒ Animals and other organisms
☒	☐ Clinical data
☒	☐ Dual use research of concern
☒	☐ Plants

Methods

n/a	Involved in the study
☒	☐ ChIP-seq
☒	☐ Flow cytometry
☒	☐ MRI-based neuroimaging

Animals and other research organisms

Policy information about studies involving animals; ARRIVE guidelines recommended for reporting animal research, and Sex and Gender in Research

Laboratory animals	Not applicable
Wild animals	Not applicable
Reporting on sex	Female samples
Field-collected samples	Sample collected from farmed animals
Ethics oversight	As per guidelines set forth by the Committee for the Purpose of Control and Supervision on Experiments on Animals (CPCSEA), India, with the Institutional Animal Ethics Committee (IAEC) approval.

Note that full information on the approval of the study protocol must also be provided in the manuscript.

Plants

Seed stocks	Not relevant
Novel plant genotypes	Not relevant
Authentication	Not relevant
